# Incidence and outcome of 18-fluorodeoxyglucose positron emission tomography/computed tomography-detected breast lesions

**DOI:** 10.1186/bcr2961

**Published:** 2011-11-04

**Authors:** LS Haine, G Rutherford, CE Ingram, IJ Jolley, O Hatsiopoulou

**Affiliations:** 1 The Royal Hallamshire Hospital, Sheffield, UK

## Introduction

Breast cancer accounts for around 16% of female deaths in the UK. Usual diagnosis is via the symptomatic pathway or screening. Positron emission tomography/computed tomography (PET/CT) is a modality with increasing applications in staging malignancies and investigating symptoms. It increasingly detects incidental breast lesions. The aim of this study was to evaluate the incidence and outcome of PET/CT-detected breast abnormalities.

## Methods

The radiology information server (CRIS) was interrogated to produce a list of PET/CT scan results containing the word 'breast' over 4 years. Scans performed on patients with known breast malignancy were excluded. A further CRIS search was used to determine if subsequent breast imaging was performed. Pathology was obtained and hospital notes were reviewed.

## Results

Thirty patients were found to have incidental breast lesions on PET/CT scanning. There were 19 masses, seven areas of focal fluorodeoxyglucose (FDG) uptake, two areas of calcification, one case of asymmetry and one area of thickening. In total, 16/30 patients underwent breast assessment (11 masses, four focal increased uptake and one bilateral calcification). Out of 11 patients with masses, six were proven to have corresponding invasive cancers and two had ductal carcinoma *in situ*. The remaining three were normal or had benign disease. Of the four patients with focal increased FDG uptake, one patient had invasive cancer, one had a fibroadenoma and two were normal. The bilateral calcifications corresponded to lymph nodes.

## Conclusion

The incidental detection of breast malignancy by PET/CT was significant at 56%. Breast assessment should be performed in this population.

